# Live imaging approach of dynamic multicellular responses in ERK signaling during vertebrate tissue development

**DOI:** 10.1042/BCJ20210557

**Published:** 2022-01-20

**Authors:** Tsuyoshi Hirashima

**Affiliations:** 1 The Hakubi Center, Kyoto University, Kyoto, Japan; 2 Laboratory of Bioimaging and Cell Signaling, Graduate School of Biostudies, Kyoto University, Kyoto, Japan; 3 Japan Science and Technology Agency, PRESTO, Kawaguchi, Japan

**Keywords:** ERK map kinase, live imaging, multicellular response, tissue development

## Abstract

The chemical and mechanical responses of cells via the exchange of information during growth and development result in the formation of biological tissues. Information processing within the cells through the signaling pathways and networks inherent to the constituent cells has been well-studied. However, the cell signaling mechanisms responsible for generating dynamic multicellular responses in developing tissues remain unclear. Here, I review the dynamic multicellular response systems during the development and growth of vertebrate tissues based on the extracellular signal-regulated kinase (ERK) pathway. First, an overview of the function of the ERK signaling network in cells is provided, followed by descriptions of biosensors essential for live imaging of the quantification of ERK activity in tissues. Then adducing four examples, I highlight the contribution of live imaging techniques for studying the involvement of spatio-temporal patterns of ERK activity change in tissue development and growth. In addition, theoretical implications of ERK signaling are also discussed from the viewpoint of dynamic systems. This review might help in understanding ERK-mediated dynamic multicellular responses and tissue morphogenesis.

## Introduction

During the developmental processes of multicellular organisms, tissues and organs are spontaneously organized through the exchange of information among the constituent cells. Each cell possesses multiple signaling cascades that are composed of conserved molecular parts of signal reception and transduction. Consequently, various outputs are produced from the cell to the tissue level. This leads to the generation of complex biological architectures and spatio-temporal patterns in tissue development. Despite many efforts, the integration of cell signaling systems to coordinate collective cell behavior is not fully understood. How are the signaling systems regulated in individual cells of multicellular tissues? What functions does the output via the multicellular signaling systems provide to the developing tissues?

From the viewpoint of cell signaling, developing tissues and organs are regulated by a relatively small set of paracrine factors and their downstream signal transduction cascades. The paracrine factors as ligands bind to receptors on the cell membrane, initiating a series of enzymatic reactions within the cell. This signaling response leads to the regulation of transcription factors and cytoskeletons that induce various cellular events. Most of the paracrine factors can be classified into one of the following four major families based on their structure: fibroblast growth factor (FGF) family, hedgehog family, Wnt family, and TGF-beta superfamily [1]. In this review, I focus on the FGF family, the receptor of which — a receptor tyrosine kinase (RTK) — undergoes a conformational change upon binding to the ligand, resulting in the exposure of cytoplasmic domains where ATP is used to phosphorylate specific tyrosine residues of particular proteins [2]. The RTK signal transduction pathways are widely used in multicellular tissues during developmental processes.

As a representative downstream signaling pathway associated with RTK activation, I review the multicellular responses of the extracellular signal-regulated kinase (ERK) signaling pathway in the context of vertebrate tissue development. In particular, the dynamic aspects of the ERK signaling systems in tissues, which have been uncovered owing to the recent developments in biosensor tools and live imaging techniques, will be discussed. First, a synopsis of ERK signaling systems is provided, followed by an overview of genetically encoded biosensor probes for the live imaging of ERK activity. Then, the role of ERK in various developing and growing vertebrate tissues with four examples, based on recent advances in live imaging analysis, is discussed. This review provides the current state of knowledge and fills the gaps that remain unexplored in previous reviews on ERK signaling pathways in development [3,4]. Throughout this review, protein symbols I have used the first letter uppercase for the zebrafish and all uppercase for the mammalian species (the exception being ERK).

## ERK signaling from single cells to tissues

### RTK-ERK signaling pathway

**Figure 1.**
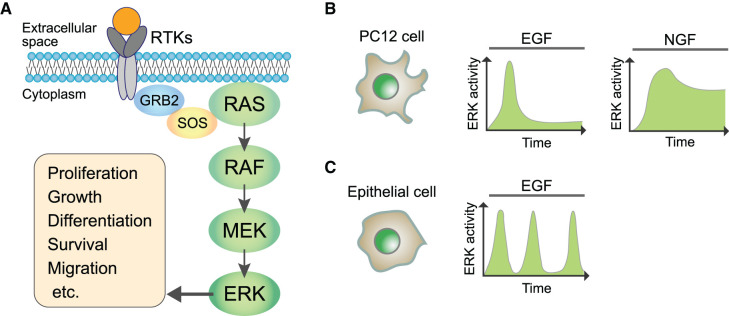
ERK signaling pathway and response. (**A**) Simplified schematic of the RTKs-ERK signaling pathway. Activation of RTKs stimulates the small GTP-binding protein RAS via the GRB2-SOS complex, triggering a series of phosphorylations of the downstream kinases RAF, MEK, and ERK. As such, extracellular chemical and mechanical factors induce ERK activation. Activated ERK phosphorylates effector proteins and regulates gene expression in the nucleus, which leads to the regulation of various cell behaviors. (**B**) Different responses of the ERK activity depending on the ligands in the PC12 cell line. ERK activation shows a transient peak upon EGF stimulation but sustained output upon NGF stimulation. (**C**) ERK activity shows oscillation or frequent pulses in response to EGF stimulation in some epithelial cell lines.

ERK is a member of the mitogen-activated protein (MAP) kinase family that responds to extracellular stimuli and is known as the classical MAP kinase [5]. In vertebrates, there are two highly homologous isoforms of ERK, ERK1 and ERK2. Both ERKs are activated more or less by all RTKs and phosphorylate the same substrates with only a modest difference in efficiency [5]. In eukaryotic cells, RTKs utilize a highly conserved signal transduction pathway in which the signal is transmitted by the binding of RTKs to growth factors, including FGF, epidermal growth factor (EGF), and vascular endothelial growth factor (VEGF) [6–8]. In many cases, two receptors dimerize in response to ligand binding, and the signal is delivered to enhance the activity of the cytoplasmic tyrosine kinase domain. Phosphorylated RTKs then bind to the adapter protein GRB2, leading to the recruitment of the guanine nucleotide exchange factor SOS and the subsequent formation of the GRB2-SOS complex. A small GTP-binding protein RAS, anchored to the cell membrane, interacts with the GRB2-SOS complex and is converted to its active state by exchanging GDP for GTP. Active RAS plays a pivotal role as a signaling switch with respect to triggering the sequential phosphorylation of RAF and MEK [9,10]. Both ERK1 and ERK2 are then activated by the dual-specificity kinase MEK1/2 through the phosphorylation of threonine and tyrosine amino acid residues of ERK1/2. Activated ERK is known to regulate various phenomenon, including cell proliferation, growth, differentiation, survival, death, and migration [5,11,12] (Figure 1A).

ERK signaling is one of the most prominent pathways underlying various physiological functions, and its activation mechanisms are diverse. The signals may be triggered by chemical ligands, mechanical forces, or the rigidity of the extracellular environments. In several mammalian cells and tissues, ERK activation is induced by mechanical cell stretch, regardless of whether the process is cyclic or not [13–17]. For example, in Madin-Darby canine kidney (MDCK) cells, transient autophosphorylation of EGFR occurs with its peak at 1 min after cell stretch [14]. In adult human skin cells, stress fibers were proposed to be a tension sensor for ERK activation, owing to the positive correlation between ERK activation on stress fibers and tensile forces acting on the fibers [18]. Moreover, stretch-induced ERK activation has been observed in Drosophila pupal tissues [19,20]. In addition, EGFRs have been implicated in integrin-dependent sensing of extracellular matrix rigidity, demonstrating the interactions between RTKs and physical microenvironments [21].

### Dynamic responses of ERK activity

ERK activity shows a variety of temporal patterns in response to different kinds of growth factors at the single-cell level. As such, various parameters, including amplitude, duration, and frequency of ERK activity of the ERK signaling dynamics, are regulated to ensure different cellular functions and biological outcomes. For example, in the PC12 cell line derived from a pheochromocytoma from rat adrenal glands, ERK is transiently activated in a pulsatile manner upon stimulation by EGF, while it sustains the up-regulated state of the nerve growth factor (NGF) [22,23] (Figure 1B). In epithelial cell lines, ERK shows stable oscillations or repeated transient pulses in a stochastic manner, with its frequency being modulated by the concentration of EGF [24–26] (Figure 1C). Such responses of the ERK activity are considered to be involved in regulating cell motions in addition to specifying cell fate decisions [5,27,28].

Theoretically, the temporal dynamic patterns in the ERK activity response can be realized by mixed feedback loops, including positive and negative feedbacks, in the RTK-ERK signaling pathway [23,29–31]. The negative feedback loop is essential for oscillations of ERK activity, and multiple pathways are potentially involved at different levels mediated through receptor endocytosis, phosphorylation of upstream components, e.g. SOS and RAF, regulation of downstream transcription factors, e.g. dual-specificity phosphatases (Dusp) and Sprouty, and cross-talk regulation with other signaling axes, e.g. AKT [30,32–34]. Positive feedback also gives rise to the rapid pulse initiation of ERK activation and bistability, and appears to arise from various interactions within the RTK-ERK signaling pathway [24,25,30,35]. Although such positive and negative feedbacks are assumed in most cases, it has been shown that the RTK-ERK signaling cascade is capable of generating oscillations and bistability even without the explicit feedback loops [36]. Furthermore, more complex dynamic behaviors, including chaotic oscillations, can be generated in the simple reaction system composed of a set of kinases and phosphatases and two substrates with two modifications [37].

As such, combinations of theoretical and practical experiments have revealed the mechanism by which mixed feedbacks produce a variety of ERK activity dynamics as a network module of the RTK-ERK signaling pathway. However, in addition to internal feedback, other factors external to the ERK pathway are involved in the regulation of ERK activity. Earlier studies have indicated that ERK dynamics may be modulated by cell-to-cell interactions. For example, the frequency of ERK activity pulses, which regulate the cell proliferation rates, displayed a bell-shaped response for cell density in normal rat kidney epithelial cells [25]. This suggests that ERK activity dynamics may be regulated through intercellular communications, including paracrine and mechanical signaling.

### Genetic deficiency of ERK during embryonic development

Vertebrate ERKs are well conserved across various species, with ERK1 and ERK2 showing 83% identity [38]. It is well known that ERK1 and ERK2 may function integrally during early development and in the adult phase under various physiologically relevant conditions. Although the ERK signaling pathway has been intensively studied, the functions of ERK1 and ERK2 and their engagement in physiological events, are not fully understood. Over the past two decades, much effort has been made using mouse models to reveal the functions of ERK1 and ERK2. One such effort is the use of ERK-knockout mice. In a previous study, during development, ERK1-null mice appeared viable and showed no gross phenotype [39]. However, ERK2-null mice exhibited embryonic lethality after the implantation stage (embryonic day 6), which may have occurred because of strain-specificity or the design of the knockout allele [40–42]. As ERK2-null animals are embryonic lethal, it is essential to incorporate a conditional genetic strategy to examine the role of ERK signaling in specific cell types *in vivo* [43,44]. In another study, Frémin et al. [45] reported that ERK1 overexpression in transgenic mice rescued the embryonic phenotypes of ERK2-null mice, a result that demonstrated the functional redundancy of ERK1 and ERK2 during mouse development. These genetic studies have revealed that ERK1 and ERK2 are essential elements for the development of multicellular organisms. In addition to the insights obtained from genetic studies, imaging approaches are required to better understand the dynamic aspects of ERK signaling.

## Biosensors for live-cell imaging

Live-cell imaging is a powerful approach to evaluate spatio-temporal information regarding biophysical parameters, including molecular activities and cellular behaviors, and provide insights into interactions between the components. For quantitative analysis, various tools and techniques from different disciplines have been used, such as fluorescence probes [46], reporter animals [47], digital image processing, and data analysis [48,49]. In this section, I highlight the use of genetically encoded fluorescence probes to monitor ERK activity in cells as key tools for live imaging.

Researchers have developed several different strategies to monitor ERK activity in living cells, most of which depend on the engineering of various fluorescent proteins [3,50–53]. Genetically encoded reporter proteins enable us to repeatedly measure signal activity in live cells under less harmful conditions. In addition, stable expression allows the collection of higher quality data by ensuring steady expression levels in a high percentage of the cells in the experiments. Here, I briefly introduce two recent approaches for studying vertebrate developmental processes, one is based on Förster/fluorescence resonance energy transfer (FRET) and the other on the kinase translocation reporter (KTR). Databases of various genetically encoded biosensors for live-cell imaging have been published [54,55].

### FRET-based probes

FRET is a phenomenon by which energy is transferred from the excited state of a chromophore (donor) to another chromophore (acceptor) in the absence of photon emission and reabsorption. When the donor and acceptor are typically positioned within several nanometers of each other, energy transfer occurs; i.e. excitation of the donor results in acceptor fluorescence, if the acceptor is a fluorophore [56]. A cyan fluorescence protein variant and a yellow fluorescence protein variant are often used as the donor and acceptor, respectively, for FRET-based genetically encoded biosensors [57]. Guidelines on the experimental design of these FRET biosensors have been reported [58].

**Figure 2.**
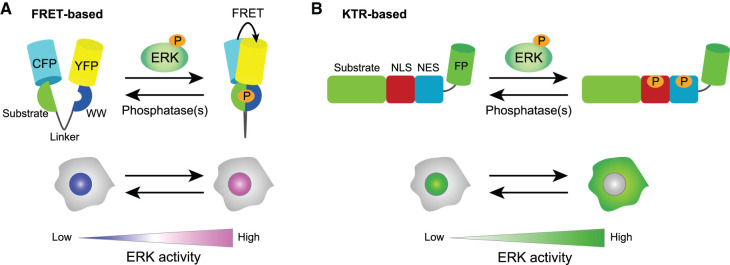
Fluorescent biosensors for ERK activity. (**A**) Schematics of the EKAREV-based FRET biosensor (upper) and the probe distribution in a cell (below). The ERAREV comprises a CFP variant, a YFP variant, a substrate peptide domain, a linker, and the WW-phosphopeptide binding. Subcellular localization of the fluorescent proteins depends on localization signals, including the NLS or the NES, in the probe. The bottom panel shows a case with the NLS-type FRET biosensor. (**B**) Schematics of the KTR-based biosensor (upper) and the probe distribution in a cell (below). The KTR comprises a fluorescent protein, a substrate recognition motif domain, the NLS, and the NES. The FP-fused KTR is localized in the cytoplasm when the ERK is active while it is in the nucleus in the case of inactive ERK.

Most FRET-based biosensors for ERK activity are based on the extracellular signal-regulated kinase activity reporter (EKAR) developed by Harvey et al. [59]. EKAR is composed of two fluorescent proteins, a specific substrate of ERK, and a domain that recognizes and binds to this substrate when phosphorylated. Komatsu et al. further developed the optimized backbone of EKAR, called EKAREV, by improving the linker through length modification for FRET live-cell imaging [60] (Figure 2A). This improvement significantly contributed to the increased dynamic range and the detection of small variations in FRET signals by intensity-based ratiometric imaging. However, a disadvantage of EKAR and EKAREV is that both respond to cyclin-dependent kinase 1 (CDK1) as well as ERK, a phenomenon that results in an increase in FRET signals during mitosis, regardless of ERK activation. Recently progress has been made to overcome this by modifying or replacing the substrate peptide to exclude cross-reactivity with CDK1 [61,62]. Recently, two modified EKAR biosensors without dependence on CDK1 have been reported [61,62].

The EKAREV-based FRET biosensor has been widely used to investigate multicellular responses in a variety of model organisms, including *Drosophila* [63,64], zebrafish [65,66], and mice [67–71]. Matsuda and colleagues at Kyoto University first generated transgenic mice expressing the ERK-FRET biosensors in 2012 [72]. In 2018, they further developed ‘hyBRET' in mice that work as a bioluminescence resonance energy transfer (BRET) biosensor without losing any properties of the original FRET biosensors [73]. Moreover, knock-in reporter mice of the Cre-inducible ERK-FRET biosensor from the ROSA26 locus were also generated [74]. A database that includes various mouse strains in addition to ERK-FRET mice is available [75].

Two methods to detect FRET of biosensors are available. One is a ratiometry method, based on calculating the ratio of acceptor and donor fluorescence in each pixel as a simple measure of the relative FRET efficiency. This method has been widely used to quantify FRET owing to its simplicity. However, quantitative imaging becomes challenging for light scattering tissues as wavelength-dependent scattering affects the detected ratio [59,76]. The other is the fluorescence lifetime imaging (FLIM) method. Basically, FRET efficiency can be quantified by measuring the fluorescence lifetime, the elapsed time between donor excitation and emission. FLIM has benefits with respect to quantitative imaging of light scattering tissues as it does not depend on changes in probe concentration and excitation intensity in either one-photon or two-photon microscopy. The disadvantage is that FLIM requires more specialized devices and analytical settings compared with the intensity-based ratiometry method [76,77].

### KTR-based probes

KTR technology allows reflecting kinase activity as nucleocytoplasmic shuttling events that can be visualized at the single-cell level. The original ERK-KTR biosensors consist of a fluorescent protein fused to a substrate peptide, which contains a nuclear localization sequence (NLS), a nuclear export sequence (NES), and an ERK phosphorylation motif (Figure 2B). The phosphorylation suppresses the effect of the NLS and enhances that of the NES, leading to the export of the fluorescent protein from the nucleus to the cytosol. That is, when the target kinase is inactive, the KTR is localized mostly in the nucleus, and when the kinase is active, the KTR is mostly found in the cytoplasm. Thus, the nucleocytoplasmic ratio of the fluorescence intensity in the image enables the quantitative measurement of dynamic kinase activity in the individual cells. Since the KTR requires only one type of fluorescent protein while FRET requires two, it is more suitable for multiplexed fluorescence imaging [78,79].

Recently, the KTR-based ERK biosensor has been used to monitor the ERK activity dynamics in individual cells of various model organisms and biological phenomena. Gagliardi et al. [80]expressed the KTR-based biosensor in a human breast epithelial cell line and demonstrated that apoptotic cells produce ERK/Akt waves propagating into their neighboring cells to provide local survival signals. de la Cova et al. [81] measured the dynamics of ERK activity in the vulval precursor cells of *Caenorhabditis elegans*, and found that the cell fate decision was regulated through the frequency of ERK activity pulses. In another study, Okuda et al. generated a transgenic line of zebrafish in which ERK-KTR was expressed specifically in vascular endothelial cells. This revealed that endothelial ERK signaling is critical for the development of arterial vessels as well as the patterning of connections between arteries and veins [82]. Moreover, two groups reported transgenic mice expressing ERK-KTR to assess ERK activity and cell fate decisions during mouse preimplantation development [83,84].

## Biological examples

Here, I will introduce four examples of vertebrate development and regeneration from studies using genetically encoded biosensors for ERK activity. These show novel biological implications with fundamental concepts of dynamic systems and systems biology.

### Cochlear duct elongation — ERK propagation via mechanical diffusion

A typical example of a spiral-shaped organ in our body is the mammalian cochlear duct, which is a tonotopically organized auditory organ in the inner ear [85,86]. During the developmental process in mouse embryos, the cochlear duct elongates, bends, and coils to form a characteristic spiral shape. Previous genetic studies have clarified that elongation of the cochlear duct requires FGF signaling in the epithelial cells [87–90]. However, it is unclear how FGF signaling regulates cellular and tissue behaviors during cochlear morphogenesis.

**Figure 3.**
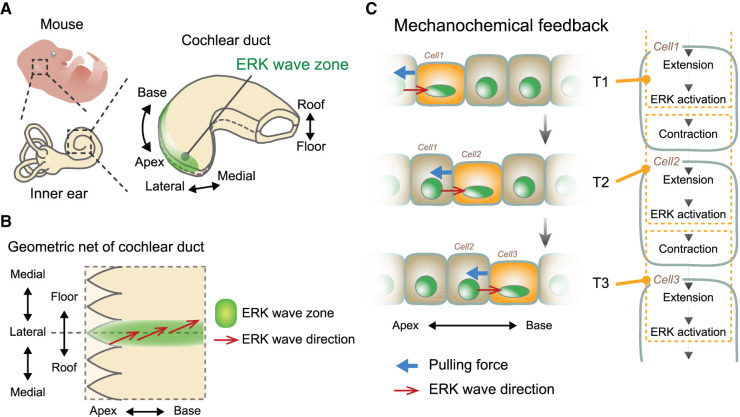
ERK activity propagation during mouse cochlear duct elongation. (**A**) Anatomical structure of the murine cochlear duct of the inner ear. Green region represents the ERK wave zone in which the ERK activity propagates intercellularly. (**B**) Geometric net of the cochlear duct. Red arrows represent the ERK wave direction. The oscillatory ERK activity propagates from the roof-apex to the floor-base on the lateral side of the elongating cochlear duct. Cells flow in the opposite direction of the ERK wave (not depicted). (**C**) A schematic of mechanochemical feedbacks that enable ERK activity wave propagations. Owing to pulling forces, an extension of Cell1 triggers EGFR-dependent ERK activation at time point 1 (T1, upper). ERK activation induces contraction in Cell1, generating a pulling force on Cell2, leading to the induction of extension-induced ERK activation in Cell2 at T2 (middle). Repeated mechanoresponsive ERK activation and ERK-mediated active contraction eventually generate ERK activity waves after T3. ERK activation occurs across multiple cells in a single snapshot, but it is represented as though ERK activation occurs in one cell at a time and propagates one after another for clarity.

Ishii et al. examined the impact of ERK signaling as the downstream effector of FGF signaling in cochlear duct development [91]. We established a novel explant culture method that facilitates 3D organ-scale long-term imaging for continuous microscopic observation during ductal outgrowth. Using the ERK-FRET biosensor mice and two-photon microscopy, we observed that the traveling waves of ERK activation propagated multiple times from the ductal tip of the roof-apex to the floor-base on the lateral side of the elongating cochlear duct (Figure 3A,B). Moreover, live-cell imaging revealed multicellular flows driven by an active cellular contraction in a direction opposite to that of the ERK activation waves. As such, advection-based elongation was proposed as the mechanism for the development of the cochlear duct [91,92]. Although previously observed in MDCK cells and adult mouse skins, this was the first study to demonstrate opposite ERK activity waves and multicellular flows during the morphogenetic processes in developing mammalian organs [14,67,93,94].

To date, the proposed mechanisms underlying the cell-to-cell propagation of ERK activity are classified in terms of spatial mediators involving either mechanical forces or ligand diffusion. Quantification of tissue velocities and change in ERK activity from the time-lapse imaging data of the cochlear duct revealed that ERK activation occurs after local tissue expansion along the apex-base axis [91]. This was also observed in MDCK cells under confluent conditions [14]. Moreover, both ERK waves and cellular flows were significantly dampened when actomyosin-dependent contraction was reduced, indicating the necessity of mechanical contractile forces. In addition to this hypothesis, the propagation mechanism may be explained by ERK-mediated mechanochemical feedback in epithelial tissues. To elaborate, ERK activation by cell stretch triggers cellular contraction, pulling the subsequent follower cells and eventually generating the propagation of ERK activation [14,91,95] (Figure 3C). This mechanism is reflected in bulk cells of the tissues and relies on the boundary condition of cell collectives as an initiator of ERK traveling waves. As such, cells in the duct apex are assumed to exert a constant pulling force on the neighboring follower cells, generating oscillatory traveling waves of ERK activity; however, the details of the mechanism by which ERK activation occurs in the boundary cells remain unclear. Despite this, it is established that the mechanical coupling of cells physically connected via cell adhesion results in mechanical diffusion, which mediates intercellular signal transmission [95]. Notably, this proposal does not assume diffusion in extracellular spaces or association with neighboring growth factors for intercellular transmission, unlike the ligand-diffusion-based mechanism. Although mechanochemical feedback can explain the experimental results of cochlear duct development and MDCK collective cell migration assay, the ligand diffusion‒based mechanism may also be involved, as explained in the next section.

### Scale bone regeneration — ERK propagation via ligand diffusion

**Figure 4.**
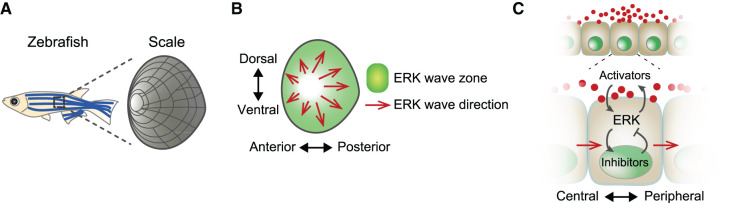
ERK activity propagation during zebrafish scale bone regeneration. (**A**) Schematic of a zebrafish scale. (**B**) ERK wave directions in the osteoblasts of the scale are shown in red. Green region represents the ERK wave zone. The ERK wave is propagated from the source region in the center of the scale (white). (**C**) Schematics of feedback systems enabling excitable wave propagation. ERK activity is amplified through a positive feedback loop between ERK and a chemical ligand activator and is suppressed by a negative feedback loop via inhibitors. ERK activity wave propagation is enabled owing to the diffusion of activators in the extracellular space.

A ligand diffusion‒based mechanism was proposed in the scale bone regeneration of adult zebrafish. Zebrafish scales have a disc-shaped structure in which osteoblasts are arranged next to the bone to form a protective shield (Figure 4A). De Simone et al. [96] reported that ERK activity propagates across osteoblasts during the regeneration of zebrafish scales. Following the construction of a transgenic ERK-KTR reporter line, they found that the oscillatory concentric traveling waves of ERK activity were generated from the center of the regenerating scale, which regulates cellular extension, contraction, and bone growth (Figure 4B). *In vivo* live imaging for 2 weeks revealed the spatio-temporal dynamic patterns of ERK activity, which traveled across several hundred scales over a period of 2 days.

The proposed mechanism relies on the excitability of the cells due to a response to weak external stimuli and the diffusion of ligands, which then mediate signal transmission from one cell to another. According to this mechanism, ERK activity stimulated by a chemical ligand would be amplified even with a weak stimulus through a positive feedback loop between ERK and the diffusible ligand (Figure 4C). With this excitability and ligand diffusion, the ERK signal can be transmitted cell-to-cell as traveling waves. Once ERK activation is initiated, delayed self-inhibiting negative feedback occurs through Dusp and Sprouty proteins that inhibit the ERK signaling cascade. This shapes the distribution of ERK activation into a peak form (Figure 4C). This was confirmed following transcriptome analysis, which revealed that the expression levels of dusp5 and spry4 in osteoblasts correlated with the activation or deactivation of ERK activity, indicating their potential inhibitory effects [96]. This three-variable excitable system, including positive and negative feedback via ERK, was formalized as a mathematical model in a reaction-diffusion scheme. The simulation results thoroughly explained the ERK activity waves observed during the regeneration of zebrafish scale bone [96,97].

Chemical diffusion-based wave propagation is considered to exist in various other systems, such as skeletal and nervous tissues, and would be a common framework for the transmission of biological information [98,99]. Indeed, it enables the achievement of long-range transmission of signals even at the millimeter scale without the loss of signal amplitude [96,97]. However, to enable robust spatial information transfer, it is essential to ensure that the microenvironments of extracellular spaces be stably maintained to avoid disturbances during ligand diffusion, as these transmissions are susceptible to extracellular environments. In contrast, the force-mediated intercellular signal transmission mentioned previously is less affected by the extracellular conditions but is sensitive to variations in physical cell states, including mechanical properties and active force generation. As both mechanisms included a broad class of reaction-diffusion systems, the mechanism of signal transmission may depend on the biological context.

### Somitogenesis — noise resistance and size scaling

Somitogenesis is a segmentation process that occurs during vertebrate development, in which the presomitic mesoderm (PSM) becomes an organized structure of segmented blocks called somites. Somites are repeated structures that eventually give rise to mature tissues, including bones and muscles of the trunk and tail of segmented animals [100]. Previous genetic and imaging studies have demonstrated that somitogenesis is regulated by the oscillatory expression of biological clock genes, such as her1 and her7 in zebrafish and *Hes7* in mice [101–104]. The core component of the her/Hes gene circuits includes an auto-regulated negative feedback loop that generates the temporal rhythm of segmentation clocks during development [105,106]. Moreover, her/Hes induces coupled oscillations of FGF signaling that produce gradients from the posterior to the anterior in the PSM, which in turn, regulate the transcription of her/Hes. This indicates that her/Hes and FGF signaling form the oscillatory networks [107]. In addition, the lack of clock genes in mice and zebrafish results in aberrant spatial patterns of somites with irregular sizes [103,108], suggesting that regulatory networks of clock genes play a role in the size regulation of segments. Based on the images of fixed samples, it has been proposed that the FGF-ERK signaling axis reflects the gradient of FGF in the PSM, a phenomenon that controls the segmentation process [109,110] and thus lacks the dynamicity associated with ERK activity during somitogenesis.

The first report on the live imaging of ERK activity during somitogenesis involved zebrafish embryos [66]. After injecting mRNAs encoded with the ERK-FRET biosensor into the yolk of zebrafish embryos at the single-cell stage, FGF-dependent ERK activity dynamics were observed in live embryos. Subsequently, a transgenic zebrafish line was generated [111], which facilitated the examination of the spatio-temporal patterns of ERK activity in zebrafish using fluorescence live imaging.

In these two studies, the live imaging technique aided the discovery of the role of ERK activity in somitogenesis. Sari et al. considered noise resistance in their study, and the ERK reporter in zebrafish embryos was used to examine the spatio-temporal patterns of ERK activity in PSM. They observed a sharp boundary of the ERK activity along the antero-posterior axis, corresponding to the prepattern of segments [66]. Live imaging revealed that the boundary shifted toward the posterior side in a stepwise manner during somitogenesis. Notably, the sharp border of the ERK activity was generated in the posterior PSM as normal, even when both her1 and her7 were knocked down. This indicated that the segmentation clock factors do not affect the formation of the shape boundary of ERK activity in the PSM. Through imaging and quantitative analysis, it was proposed that the formation of irregularly sized somites in clock-deficient zebrafish could be attributed to the temporal irregularity of the stepwise shift of ERK activity in the PSM [66]. This was supported by a follow-up mathematical model that explained the mapping from the FGF gradient to the shape boundary of ERK activity, by assuming the bistability of the ERK signaling networks introduced by hypothetical positive feedback loops in the system [112]. This computational analysis explained the temporal regulation of the ERK activity shift in a stepwise manner. In addition, a noise-resistant mechanism using bifurcation diagrams for the robust size regulation of somitogenesis was also elucidated, shedding light on an unexplored aspect of somitogenesis.

The second study considered the effect of tissue size scaling. The size of developing embryos varies even in the same species. However, in most cases, the tissue is scaled according to the body size. In the case of the zebrafish, the size of developing somites was reported to scale with the PSM length in a linear manner [113]. However, the mechanism of somite scaling is unclear. To further understand this process, Ishimatsu et al. decreased the embryo size by surgically removing cells at the blastula stage, creating small embryos in which the number of somites was the same, but the size was smaller than that of the control embryos [65]. They then used the ERK-FRET biosensor in the zebrafish embryos to quantify ERK activity as an effector of the FGF gradient in the PSM. Interestingly, they found that the spatial pattern of ERK activity corresponded to the PSM length even in the surgically reduced embryos. This indicated that the spatial distribution of FGF-ERK signaling may be attributed to somite scaling. Finally, the insights obtained from the experimental data led to the idea that the ‘clock and scaled gradient model' can theoretically explain somite scaling. Altogether, these two arguments are key to improving our understanding of the mechanism by which ERK robustly regulates the spontaneous organization of vertebrate morphology. However, the mechanistic understanding of the role of ERK activity in somitogenesis remains far from complete.

### Tracheal chondrogenesis — incoherent feed-forward loops

**Figure 5.**
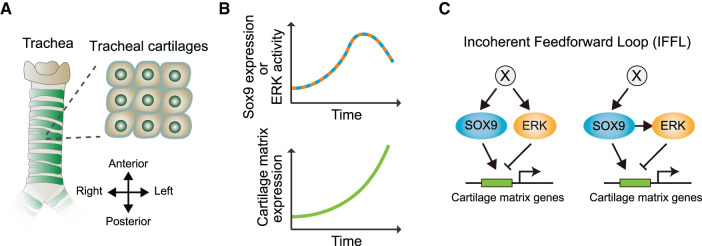
SOX9-ERK regulation in murine tracheal cartilage development. (**A**) Anatomical structure of mammalian trachea. Tracheal cartilages are shown in green. (**B**) Output level as a function of time. Sox9 expression level and ERK activity show a temporal peak (upper), and cartilage matrix expression level shows a monotonic increase. (**C**) Potential regulator networks that make up incoherent feed-forward loops. Cartilage matrix genes, such as Col2a1 and Agc1, are positively regulated by SOX9 and negatively regulated by ERK. On the left, an undetermined factor X that is a common upstream regulator in SOX9 and ERK, is presented, and on the right, X is shown to regulate SOX9 directly, but not ERK, and SOX9 is shown to control ERK.

The mammalian trachea is a tubular respiratory system that is composed of different types of tissues, such as cartilage and epithelium [114,115]. The tracheal cartilage is formed on the ventral side of the epithelial airway through mesenchymal aggregation, which forms a semi-ring during development (Figure 5A). The development of tracheal cartilages has been studied primarily using mouse genetics, which has uncovered the role of multiple transcription factors and signaling pathways. A critical transcriptional factor is the master regulator SRY-box transcription factor 9 (Sox9), which is regulated by Sonic Hedgehog (Shh) [116,117] and induces the expression of several cartilage matrix molecules, including collagen II alpha 1 (Col2a1) and aggrecan (Agc1) [118,119]. The importance of Sox9 in tracheal development was clearly demonstrated in mesenchyme-specific *Sox9* knockout mice that exhibit a complete absence of the trachea [120,121]. Genetic studies have also revealed the importance of the FGF-ERK signaling axis in tracheal cartilage development. Ubiquitous knockout of *Fgf10* and its main receptor in the mesenchyme causes severe malformations of the tracheal cartilages [122,123]. In addition, compound knockout of *Mek1* and *Mek2* in the tracheal mesenchyme resulted in the loss of ERK activity and tracheal chondrogenesis [43]. These studies have demonstrated the necessity of SOX9 and FGF-ERK signaling in normal tracheal tissue. However, the mechanism by which these factors are integrated to regulate tracheal chondrogenesis remains unclear.

To examine the mechanism by which the Sox9 and ERK signaling pathways play a role in tracheal cartilage development, Yoshida et al. utilized fluorescence reporter mice using two-photon microscopy [124]. In this study, Sox9 expression and ERK activity were quantified using Sox9-EGFP knock-in mice, in which the expression level of EGFP correlated with the endogenous expression of *Sox9* [125,126] in ERK-FRET biosensor-expressing mice [73], respectively. Volumetric live imaging throughout the differentiation of murine trachea cartilage revealed that both Sox9 expression and ERK activity exhibited non-monotonic profiles over time, with a peak at embryonic day 14.5, whereas the expression levels of their downstream cartilage matrix genes monotonically increased (Figure 5B). In addition, inhibitor assays revealed that ERK signaling reduced the expression level of cartilage matrix genes during this period, indicating that the cartilage matrix genes are negatively regulated by ERK activity and positively regulated by Sox9. As Sox9 expression and ERK activity both peak at the same time, it is speculated that either they have a common upstream factor X or the upstream factor of Sox9, such as Shh, thereby indirectly affecting ERK activity through Sox9 expression. In either case, the input–output paths via Sox9 expression and ERK activity are opposing and ultimately lead to antagonistic effects (Figure 5C). This is referred to as the incoherent feed-forward loop (IFFL) [127,128]. The IFFL regulatory circuit is known as a network motif that provides a biphasic steady-state response to the input level and is frequently found in biochemical regulatory systems [129].

The IFFL is a typical set of recurring circuits evolutionarily conserved across species and has various physiological functions. The well-known functions of IFFL in the context of dynamic information processing are pulse generation and response acceleration caused by a step input stimulus [128,129]. Moreover, IFFL may also provide robustness, as proposed in the theoretical analysis of tracheal chondrogenesis [124]. To examine the role of ERK as a negative regulator, a hypothetical pathway that includes only the positive regulation of Sox9 in the cartilage matrix gene without the regulation of ERK activity was considered. A comparison between IFFL and the hypothetical pathway reveals the benefit of having ERK as a core regulator in tracheal development. Sensitivity analysis of cartilage matrix gene expression in the mathematical model indicated that the use of ERK as a suppressor in the signaling pathways improves robustness (compared with the single regulatory pathway) in response to fluctuations in the upstream regulator. Tissue-scale fluorescence imaging and theoretical analysis have uncovered a partial role for ERK activity in tracheal chondrogenesis. However, further investigations are required for a full understanding of the dynamic aspect and biological functions in tissue development.

## Conclusions and discussion

I have presented four biological examples showing the roles and spatio-temporal dynamics of ERK activity in different contexts. In the first two examples, we saw that the oscillatory waves of ERK activity spread in large-scale tissues during cochlear duct development in murine embryos and scale bone regeneration in zebrafish. Despite the similarity in the dynamic patterns of ERK activity, different mechanisms have been proposed regarding the mechanism by which the ERK signal is transmitted cell-to-cell. I have also discussed possible advantages of intercellular signal transmission depending on whether a mechanical force-based or diffusible ligand-based regime is chosen. In the latter two examples, the impact of ERK activity on the robustness of systems is explained by selecting zebrafish somitogenesis and murine tracheal chondrogenesis as paradigms. In both cases, the implications were investigated by evaluating ERK activity in growing volumetric tissues by live-cell imaging along with computational and theoretical analysis of mathematical models.

Characteristic spatio-temporal patterns of ERK activity appear to be common in many tissues and organs over biological species, but little is known about why it happens. One reason would come from the inherent properties of ERK signaling pathway that can potentially satisfy diverse biological demands with its high degree of freedom as the input–output signaling system. The signaling pathway generates nonlinear input–output relationships and rich temporal dynamics through the molecular network circuits, including a chain of kinase and phosphatase reaction cycles and intricate feedbacks. Moreover, the ERK signaling system utilizes both chemical and mechanical factors as mediators, which gives rise to diversity in the spatial patterning. Therefore, the ERK signaling pathway has been adopted as a versatile cellular network device. The ERK signaling pathway is just one building block among many pathways that provide spatio-temporal information into living tissues. Further studies on interplay with other signaling systems using the live imaging approach will pave the way to a comprehensive understanding of the dynamic multicellular responses.

Overall, the live imaging approach facilitates the quantification of the dynamic cellular responses at the single-cell level in tissues. However, it is limited by a lack of resources and low throughput performance. As previously mentioned, transgenic mouse and zebrafish lines expressing genetically encoded biosensors to monitor ERK activity have been generated. As such, the sharing of information on the relevant databases will promote the efficient use of animal resources. Recent developments such as two-photon microscopy [130–132] and light-sheet microscopy [133,134] accelerate the acquisition of a large amount of image data sets with high spatio-temporal resolutions over millimeter scales. Intravital imaging of mouse embryos will also be useful for a better understanding of the *in vivo* dynamics of molecules and cells [135]. In addition, ex vivo reconstruction of the system is an alternative approach to the use of live animals. Organoid systems enable the recapitulation of higher-order functions of volumetric tissues with respect to *in vivo* tissue architectures [136,137], and compositions of various kinds of organoids, including what were dealt with in this review, have been established, such as inner ear [138], early somites [139,140], and trachea [141]. Moreover, an organs-on-a-chip strategy will supplement higher-throughput performance with microscopy analysis [142,143]. These strategies may improve the extent of throughput performance for the analysis of different samples and accelerate the understanding of ERK-mediated dynamic multicellular responses and tissue morphogenesis.

## Abbreviations

BRET: bioluminescence resonance energy transfer
CDK: cyclin-dependent kinase 1
EGF: epidermal growth factor
EKAR: extracellular signal-regulated kinase activity reporter
ERK: extracellular signal-regulated kinase
FGF: fibroblast growth factor
FLIM: fluorescence lifetime imaging
FRET: Förster/fluorescence resonance energy transfer
IFFL: incoherent feed-forward loop
KTR: kinase translocation reporter
MAP: mitogen-activated protein
MDCK: Madin-Darby canine kidney
NES: nuclear export sequence
NLS: nuclear localization sequence
PSM: presomitic mesoderm
RTK: receptor tyrosine kinase
VEGF: vascular endothelial growth factor
